# Effect of protein and carbohydrate distribution among meals on quality of life, sleep quality, inflammation, and oxidative stress in patients with type 2 diabetes: A single‐blinded randomized controlled trial

**DOI:** 10.1002/fsn3.2570

**Published:** 2021-09-13

**Authors:** Fatemeh Nouripour, Zohreh Mazloom, Mohammad Fararouei, Ali Zamani

**Affiliations:** ^1^ Department of Clinical Nutrition School of Nutrition and Food Sciences Shiraz University of Medical Sciences Shiraz Iran; ^2^ Department of Epidemiology School of Health Shiraz University of Medical Sciences Shiraz Iran; ^3^ Department of Internal medicine School of Medicine, Endocrinology and Metabolism Research Center Shiraz University of Medical Sciences Shiraz Iran

**Keywords:** evening meal, inflammation, macronutrients, oxidative stress, quality of life, sleep quality, type 2 diabetes

## Abstract

**Background/Objectives:**

Patients with diabetes mellitus have a lower quality of life and sleep compared with healthy individuals. Nutrition therapy has an important role in the management of diabetes and can improve inflammation and quality of life in patients with diabetes. The present study aimed to evaluate the effect of high‐protein versus high‐carbohydrate intake during evening meal on quality of life, sleep quality, inflammation, and oxidative stress in subjects with type 2 diabetes.

**Subjects/Methods:**

This is a 10‐week randomized controlled trial. 96 adult patients with type 2 diabetes were assigned into one of the following three groups: standard evening meal (ST), high‐carbohydrate evening meal (HC), and high‐protein evening meal (HP). The effect of these diets was examined on high‐sensitivity C‐reactive protein, malondialdehyde, quality of life, and sleep quality.

**Results:**

Sleep quality improved significantly in all groups (*p* < .05). The quality of life and high‐sensitivity C‐reactive protein improved in all groups except for the HP group (*p* < .05). Serum malondialdehyde level did not change significantly throughout the study (*p* > .05).

**Conclusions:**

Small manipulation of protein and carbohydrate distribution among the meals might not affect sleep quality. A diet with an even distribution of macronutrients among the meals or with a higher percentage of carbohydrates in the evenings can improve the quality of life and reduce inflammation in patients with type 2 diabetes, while a diet with a higher percentage of protein in the evenings may not improve it.

## Graphical abstract

A diet with an even distribution of macronutrients among the meals or with a higher percentage of carbohydrates in the evenings may improve the quality of life and inflammation in patients with type 2 diabetes. A diet with a higher percentage of protein in the evenings may not improve the quality of life and inflammation in patients with type 2 diabetes.

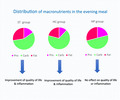

## 1 INTRODUCTION

Patients with diabetes mellitus are at increased risk of macrovascular and microvascular complications including cardiovascular diseases, nephropathy, retinopathy, and neuropathy. Diabetes is a leading cause of blindness, renal failure, lower extremity amputation, and death, which imposes a great financial burden on society and healthcare systems (van Dieren et al., 2010; Viswanathan & Sathyamurthy, 2015). Chronic inflammation has a role in the pathogenesis of diabetes complications. On the other hand, hyperglycemia induces inflammation and consequently increases the risk of cardiovascular diseases (Nowlin et al., 2012). In addition, patients with diabetes have a lower quality of life and sleep quality compared with healthy individuals (Kiadaliri et al., 2013; Luyster & Dunbar‐Jacob, 2011; Porojan et al., 2009).

Nutrition therapy has an important role in the management of diabetes and improves inflammation and quality of life in patients with diabetes (American Diabetes Association, 2016; Davis et al., 2012; Nowlin et al., 2012; Wycherley et al., 2014). In several studies, it was observed that the timing of macronutrient intake affects body weight and glycemic control (Alves et al., 2014; Sofer et al., 2011). It was also suggested that reducing carbohydrate intake during the daytime may improve inflammation in obese individuals (Sofer et al., 2011). Improvement in physical health may lead to improved quality of life (Porojan et al., 2009; Wycherley et al., 2014). In addition, the composition of the diet and the dinner meal, for example its macronutrient composition, may affect the structure and quality of sleep (Cao et al., 2016; St‐Onge et al., 2016). Sleep quality affects body weight and glycemic control (Golem et al., 2014; Lee et al., 2017). Hence, we intended to examine whether manipulating macronutrient distribution among the meals would have beneficial effects in patients with type 2 diabetes.

The aim of the present study was to evaluate the effect of high‐protein versus high‐carbohydrate intake in the evening meals on quality of life, sleep quality, inflammation, and oxidative stress in subjects with type 2 diabetes.

## 2 MATERIALS AND METHODS

### 2.1 Subjects

This study is part of a larger trial, examining the effect of protein and carbohydrates distribution among meals on health parameters of patients with type 2 diabetes. Ninety‐six patients with type 2 diabetes were recruited mainly by public advertisements. The inclusion criteria were type 2 diabetes, age 30–65 years, diabetes duration of ≤15 years, HbA_1_c ≤ 8%, body mass index ≥22 and <35 kg/m^2^, not taking insulin or α‐glucosidase inhibitors, stable weight (±3 kg) during the past 3 months, not being on weight loss or vegan diet. Subjects with hepatic, cardiac, renal, thyroid, respiratory, gastrointestinal, and eating disorders were not included. The exclusion criteria were poor compliance to the diet or change in medications use throughout the study (Nouripour et al., 2021).

### 2.2 Study design and procedure

This is a 10‐week single‐blinded, parallel, randomized controlled trial. The participants were not aware of the type of diet they were received. The primary outcome of the current study was to measure the quality of life of the participants. The secondary outcomes were to measure the participants’ sleep quality, inflammation, and oxidative stress. The study protocol was explained to the volunteers, and written informed consent form was signed by all. The demographic characteristics, dietary habits, and medical history of the participants were documented. This study was conducted according to the Declaration of Helsinki guidelines and its later amendments, which was approved by the local Ethics Committee of Shiraz University of Medical Sciences (No. IR.SUMS.REC.1397.101). The study protocol is registered in the Iranian Registry of Clinical Trials (No. IRCT20170427033666N2).

Before initiating the intervention, there was a 2‐week run‐in period (weeks −2 to 0). The participants were asked to maintain their usual lifestyle including diet, physical activity, and smoking habits during this period and refrain from unusual activities, such as fasting. They were also asked to keep a 3‐day food record (2 weekdays and 1 weekend day).

Subjects who did not violate the run‐in period protocol were randomly allocated to either of the following three groups, using block randomization: standard evening meal (ST), high‐carbohydrate evening meal (HC), and high‐protein evening meal (HP). A block size of 3 was used for the randomization.

Patients were asked not to change their physical activity level and smoking habits throughout the study. International Physical Activity Questionnaire (IPAQ) was used to measure the physical activity level of the participants (Criniere et al., 2011; Moghaddam et al., 2012).

### 2.3 Diets

The energy requirement of each subject was calculated, using the Institute of Medicine equations (Institute of Medicine, 2002). All participants received a diet composed of 15%–20% protein, 50%–55% carbohydrate, and 25%–30% fat. Exchange lists for meal planning (”Exchange Lists For Meal Planning", 2008) and dietary recommendations were provided for all subjects (Supplementary Material). The protein and carbohydrate distribution among the meals were different between the diets. In the ST group, protein and carbohydrates were rather evenly distributed among the meals. In the HC group, a higher percentage of carbohydrate (40%–45% of total carbohydrate intake) was provided at dinner and evening snack. The remaining carbohydrate (55%–60% of total daily intake) was provided in the breakfast, morning snack, lunch, and afternoon snack. In the HP group, 40%–45% of total protein intake was at dinner and evening snack. Protein and fat intake in the HC dinner and similarly carbohydrate and fat intake in the HP dinner were reduced to prevent excess energy intake in the evening. The menu of the prescribed diets is provided in Supplementary Material.

The participants were visited by the nutritionist at weeks 2, 5, and 10. They received dietary consultation at each visit and were persuaded to follow their prescribed diet. Participants were asked to fill 3‐day food records (2 weekdays and 1 weekend day) at weeks 2, 5, and 10. Nutritionist IV software (version 3.5.2 1994; N‐Squared Computing, San Bruno CA) was used to assess dietary intake and compliance. Satisfaction with the diets was assessed by means of a questionnaire at the end of the study (Nouripour et al., 2021). More details about the prescribed diets and study procedure were described previously (Nouripour et al., 2021).

### 2.4 Questionnaires

#### 2.4.1 Quality of life

Quality of life was assessed, using 36‐Item Short‐Form Health Survey version 2 (SF‐36v2) at weeks 0, 5, and 10. This questionnaire is validated for use among the Iranian population and patients with type 2 diabetes (Jacobson et al., 1994; Montazeri et al., 2005; Motamed et al., 2005). SF‐36 is a 36‐item questionnaire and composed of 8 scales including physical functioning, role‐physical, bodily pain, general health, vitality, social functioning, role‐emotional, and mental health. There is an item of health change in the questionnaire which is not used for scoring (Hays et al., 1993). The score of each scale ranges from 0 to 100, the higher the scores the better the health status. Scores of physical functioning, role‐physical, bodily pain, and general health scales are aggregated to calculate the Physical Health score. Scores of vitality, social functioning, role‐emotional, and mental health scales are aggregated to calculate the Mental Health score.

#### 2.4.2 Sleep quality

Sleep quality was assessed, using a validated questionnaire, the Pittsburg Sleep Quality Index (PSQI), at weeks 0, 5, and 10 (Farrahi Moghaddam et al., 2012). PSQI is a 19‐item questionnaire with 7 components including subjective sleep quality, sleep latency, sleep duration, habitual sleep efficiency, sleep disturbances, use of sleep medications, and daytime dysfunction. The score of these 7 components sums up to calculate the total score of PSQI (global sleep quality). PSQI score ranges from 0 to 21 with higher scores indicating lower sleep quality (Buysse et al., 1989).

### 2.5 Laboratory measurements

Blood tests were performed at the beginning and the end of the trial. After 12‐h fasting, blood samples were obtained and centrifuged. Serum samples were stored at −72°C until further analysis. High‐sensitivity C‐reactive protein (hs‐CRP) was measured by enzyme‐linked immunosorbent assay (ELISA) kit (Monobind Inc.). Malondialdehyde (MDA) was measured, using spectrophotometry method.

### 2.6 Statistical analysis

**FIGURE 1:**
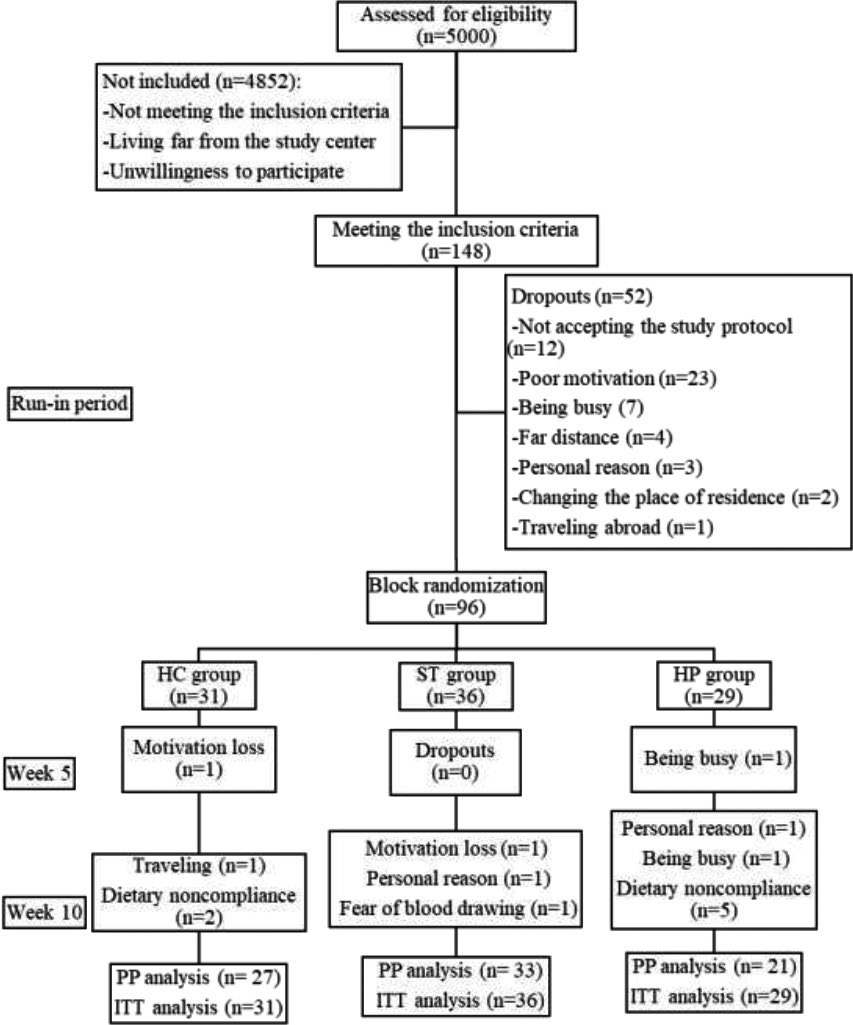
Flow diagram of the study participants

The sampling method was described before (Nouripour et al., 2021). Briefly, thirty‐six, thirty‐one, and twenty‐nine participants were randomly assigned to the ST, HC, and HP groups, respectively (Figure 1). A post hoc power analysis was conducted to measure the adequacy of the sample size in the recent analysis. According to the results, by considering quality of life as our main outcome and an α value of 0.05 and a statistical power of 80%, the statistical tests were able to detect the least significant effect on the physical health as small as 8 scores. The results are presented as mean ± standard deviation or number (percent). To compare baseline characteristics of the study participants, one‐way ANOVA and Chi‐square were used for quantitative and categorical variables, respectively. Paired *t* test and repeated measures ANOVA were used for analyzing within‐group changes. One‐way ANOVA and repeated measures ANOVA were used for analyzing between‐group differences. Correlation analysis was done using the Pearson test. A *p* value less than .05 was considered to be statically significant. SPSS version 25 (SPSS Inc.) was used for analysis. The analysis was conducted using the intention‐to‐treat (ITT) approach. The last observation carried forward (LOCF) method was used to handle the missing values. An additional analysis, using the per‐protocol (PP) approach, was also done to compare the results.

## 3 RESULTS

### 3.1 Participants and baseline characteristics

Ninety‐six participants were randomly assigned to the study groups. As it is shown in Figure 1, eight participants dropped out during the study, and 7 participants were excluded due to poor dietary compliance. Ninety‐six and eighty‐one participants were enrolled in the ITT and PP analyses, respectively. The baseline characteristics of the study participants are shown in Table 1. There were no significant differences between the groups at the baseline (*p* > .05).

**TABLE 1:** Baseline characteristics of the study participants

	Overall (*n* = 96)	ST (*n* = 36)	HC (*n* = 31)	HP (*n* = 29)	*p* value^a^
Sex					.84
Male	46 (47.9)	16 (44.4)	16 (51.6)	14 (48.3)	
Female	50 (52.1)	20 (55.6)	15 (48.4)	15 (51.7)	
Age (year)	53.8 ± 7.6	56.1 ± 7.2	54.0 ± 6.3	51.7 ± 8.2	.06
Marital status					.77
Married	90 (93.8)	33 (91.7)	30 (96.8)	27 (93.1)	
Single/Widowed	6 (6.3)	3 (8.3)	1 (3.2)	2 (6.9)	
Diabetes duration (year)	5.6 ± 4.1	5.8 ± 4.3	5.3 ± 4.3	5.5 ± 3.5	.86
Smoking (+)	6 (6.3)	1 (2.8)	2 (6.5)	3 (10.3)	.43
Glucose lowering medication (+)	87 (90.6)	33 (91.7)	28 (90.3)	26 (89.7)	.96
Weight (kg)	76.5 ± 10.7	74.7 ± 9.6	78.8 ± 12.3	76.1 ± 10.3	.30
BMI (kg/m^2^)	28.5 ± 3.4	27.8 ± 2.8	29.1 ± 4.4	28.6 ± 2.9	.30
HbA_1_c (%)	6.61 ± 0.81	6.61 ± 0.76	6.58 ± 0.81	6.64 ± 0.90	.96
hs‐CRP (µg/ml)	1.8 ± 1.5	1.8 ± 1.8	1.7 ± 1.2	1.8 ± 1.5	.08
MDA (µmol/L)	2.3 ± 0.3	2.27 ± 0.30	2.37 ± 0.29	2.30 ± 0.30	.93
Physical Health	273.8 ± 65.3	279.6 ± 64.4	262.4 ± 66.4	278.8 ± 66.1	.50
Mental Health	277.5 ± 65.5	283.3 ± 71.0	273.9 ± 71.1	277.5 ± 65.5	.80
PSQI score	7.6 ± 4.1	7.2 ± 4.3	7.8 ± 4.0	7.8 ± 4.2	.79

Note

Values are mean ± SD or number (percent).

Abbreviations: BMI, body mass index; HC, high‐carbohydrate evening meal; HP, high‐protein evening meal; hs‐CRP, high‐sensitivity C‐reactive protein; MDA, malondialdehyde; PSQI, Pittsburg Sleep Quality Index; ST, standard evening meal.

^a^ Differences between groups using one‐way ANOVA.

### 3.2 Diet and physical activity

The dietary intake of the participants at baseline is shown in Table 2. Baseline energy, carbohydrate, protein, fat, and fiber intakes of the participants had no difference between the groups (*p* > .05). Changes in dietary intakes during the study did not differ between the groups (*p* > .05, Supplementary Material). Changes in the antioxidants intake were not significant in any groups except for ascorbic acid, which increased significantly in the HC group (16.1 ± 29.7 mg, *p* = .005).

**TABLE 2:** Dietary intake of the participants at baseline

	Overall (*n* = 96)	ST (*n* = 36)	HC (*n* = 31)	HP (*n* = 29)	*p* value^a^
Energy (Kcal)	1977 ± 601	1902 ± 562	2057 ± 589	1983 ± 665	.59
Carbohydrate (g)	292.3 ± 92.0	287.8 ± 91.6	306.2 ± 94.5	282.9 ± 91.4	.59
Carbohydrate (% of energy)	59.2 ± 7.2	60.5 ± 5.6	59.5 ± 7.5	57.4 ± 8.5	.22
Protein (g)	70.7 ± 23.5	65.8 ± 20.1	73.6 ± 21.8	73.7 ± 28.5	.29
Protein (% of energy)	14.4 ± 2.1	14.0 ± 2.2	14.4 ± 1.6	14.9 ± 2.5	.19
Fat (g)	61.5 ± 24.0	57.1 ± 20.3	63.0 ± 23.7	65.4 ± 28.1	.35
Fat (% of energy)	27.9 ± 5.7	26.9 ± 5.0	27.6 ± 6.1	29.3 ± 6.1	.23
Fiber (g)	18.0 ± 8.0	15.9 ± 5.2	19.4 ± 10.0	19.0 ± 8.3	.14

Note

Values are mean ± SD.

Abbreviations: HC, high‐carbohydrate evening meal; HP, high‐protein evening meal; ST, standard evening meal.

^a^ Differences between groups using one‐way ANOVA.

Participants who received much higher or lower energy than their prescribed diets or did not follow the ratio of macronutrients in their meals were excluded from the PP analysis. Participants had no adverse effects related to the diets. Satisfaction with the diet was higher in the ST compared with the HC and HP groups.

The physical activity level of the participants at baseline and its changes throughout the study had no significant difference between the groups (*p* > .05, Supplementary Material).

### 3.3 Quality of life

Physical Health, Mental Health, and their subscales were not significantly different among the groups at baseline (*p* > .05). The effect of diets on the quality of life of the study participants is shown in Table 3. Physical Health increased significantly in ST and HC groups (*p* < .05). However, it did not change significantly in the HP group (*p* > .05). Mental Health increased significantly only in the ST group (*p* < .05). Health change improved significantly in all groups (*p* < .05). Both ITT and PP analyses revealed no significant differences between the groups regarding Physical and Mental Health (*p* > .05).

**TABLE 3:** Changes in SF‐36 questionnaire scales throughout the study

	ST (*n* = 36)				HC (*n* = 31)				HP (*n* = 29)				*p* value^b^
	Week 0	Week 5	Week 10	*p* value^a^	Week 0	Week 5	Week 10	*p* value^a^	Week 0	Week 5	Week 10	*p* value^a^	
Physical Health	279.6 ± 64.4	296.6 ± 70.9	291.8 ± 69.7	.043	262.4 ± 66.4	284.3 ± 66.6	290.4 ± 66.7	.001	278.8 ± 66.1	288.3 ± 64.7	285.8 ± 67.4	.33	.80
Physical functioning	73.3 ± 19.3	76.5 ± 17.8	77.6 ± 19.3	.11	74.5 ± 20.3	80.5 ± 18.4	79.4 ± 21.1	.051	77.6 ± 18.9	80.2 ± 16.2	78.1 ± 17.8	.38	.78
Role‐physical	67.4 ± 24.4	77.4 ± 19.8	73.3 ± 21.7	.025	66.9 ± 22.1	69.2 ± 21.5	71.0 ± 17.9	.31	70.7 ± 22.8	71.1 ± 22.6	68.5 ± 20.6	.77	.71
Bodily pain	68.1 ± 15.0	71.1 ± 19.2	72.6 ± 19.5	.17	60.3 ± 17.4	66.7 ± 16.6	69.5 ± 17.8	<.001	61.6 ± 19.5	64.1 ± 18.4	66.7 ± 19.0	.16	.63
General health	70.8 ± 23.6	71.5 ± 25.3	68.4 ± 22.9	.60	60.7 ± 26.1	68.0 ± 25.5	70.6 ± 24.0	.039	68.9 ± 24.4	72.9 ± 21.5	72.5 ± 22.1	.29	.24
Mental Health	283.3 ± 71.0	307.4 ± 66.5	300.0 ± 68.7	.022	273.9 ± 71.1	288.2 ± 59.3	288.8 ± 49.7	.089	277.5 ± 65.5	280.1 ± 65.7	279.1 ± 71.1	.71	.42
Vitality	64.6 ± 18.8	69.8 ± 19.8	69.1 ± 19.3	.007	60.1 ± 13.2	65.3 ± 16.0	64.5 ± 14.6	.036	61.4 ± 20.1	62.5 ± 17.0	62.5 ± 19.8	.86	.34
Social functioning	75.7 ± 22.1	84.0 ± 17.8	79.9 ± 21.0	.008	69.8 ± 23.9	78.2 ± 21.6	80.2 ± 18.2	.025	72.4 ± 27.0	78.4 ± 21.1	75.4 ± 21.0	.34	.57
Role‐emotional	72.2 ± 22.1	79.2 ± 19.3	76.2 ± 20.1	.053	73.9 ± 18.8	73.7 ± 22.0	73.9 ± 18.2	.99	74.4 ± 21.2	69.0 ± 24.3	71.0 ± 24.2	.34	.64
Mental health	70.8 ± 17.5	74.4 ± 16.3	74.9 ± 17.3	.044	70.2 ± 15.1	71.0 ± 15.6	71.1 ± 14.1	.73	65.7 ± 17.1	70.2 ± 18.0	70.2 ± 19.2	.009	.49
Health change	58.3 ± 23.1	61.8 ± 24.3	66.0 ± 23.3	.020	56.5 ± 22.3	62.9 ± 27.3	69.4 ± 23.9	.002	53.4 ± 25.6	58.6 ± 25.2	62.1 ± 23.7	.045	.69

Note

Values are mean ± SD.

Abbreviations: HC, high‐carbohydrate evening meal; HP, high‐protein evening meal; SF‐36, 36‐Item Short‐Form Health Survey; ST, standard evening meal.

^a^ Changes within groups using repeated measures ANOVA.

^b^ Difference between groups using repeated measures ANOVA.

### 3.4 Sleep quality

PSQI score of the participants and its components were not significantly different among the groups at baseline (*p* > .05). 61.5% of the participants had poor sleep quality (PSQI > 5). There were negative correlations between PSQI score and both Physical Health and Mental Health scores (*r* = −0.5, *p* < .001). The effect of diets on sleep quality of the study participants is shown in Table 4. Sleep quality improved significantly in all groups (*p* < .05). There were no significant differences between the groups using both ITT and PP analyses (*p* > .05).

**TABLE 4:** Changes in PSQI components scores throughout the study

	ST (*n* = 36)				HC (*n* = 31)				HP (*n* = 29)				*p* value^b^
	Week 0	Week 5	Week 10	*p* value^a^	Week 0	Week 5	Week 10	*p* value^a^	Week 0	Week 5	Week 10	*p* value^a^	
Subjective sleep quality	1.0 ± 0.8	0.8 ± 0.6	0.8 ± 0.6	.030	1.0 ± 0.7	0.9 ± 0.7	0.8 ± 0.5	.26	1.0 ± 0.7	0.8 ± 0.5	0.8 ± 0.5	.16	.95
Sleep latency	1.4 ± 1.2	1.1 ± 1.0	1.0 ± 1.1	.021	1.4 ± 1.1	1.1 ± 1.1	1.1 ± 1.0	.085	1.4 ± 1.0	1.2 ± 0.9	1.1 ± 0.9	.093	.96
Sleep duration	1.6 ± 1.0	1.5 ± 1.0	1.5 ± 1.1	.37	1.5 ± 1.1	1.5 ± 1.2	1.5 ± 1.1	.85	1.4 ± 1.0	1.2 ± 1.0	1.3 ± 0.9	.19	.68
Habitual sleep efficiency	0.7 ± 1.0	0.6 ± 1.0	0.7 ± 1.1	.79	1.1 ± 1.2	0.9 ± 1.2	0.8 ± 1.2	.24	0.9 ± 1.1	0.6 ± 1.0	0.7 ± 1.0	.088	.45
Sleep disturbances	1.4 ± 0.6	1.3 ± 0.5	1.3 ± 0.6	.20	1.5 ± 0.6	1.5 ± 0.6	1.4 ± 0.6	.27	1.5 ± 0.6	1.4 ± 0.6	1.4 ± 0.6	.36	.82
Use of sleep medications	0.4 ± 0.8	0.3 ± 0.8	0.3 ± 0.8	.28	0.5 ± 1.1	0.5 ± 1.1	0.5 ± 1.0	.82	0.6 ± 1.1	0.4 ± 0.9	0.3 ± 0.8	.11	.72
Daytime dysfunction	0.6 ± 0.7	0.6 ± 0.8	0.6 ± 0.7	.70	0.8 ± 0.8	0.6 ± 0.6	0.6 ± 0.5	.30	1.0 ± 1.0	0.8 ± 0.6	0.6 ± 0.6	.056	.38
PSQI score	7.2 ± 4.3	6.1 ± 3.5	6.2 ± 4.3	.017	7.8 ± 4.0	7.0 ± 4.0	6.6 ± 3.8	.039	7.8 ± 4.2	6.3 ± 3.4	6.2 ± 3.5	.002	.77

Note

Values are mean s± SD.

Abbreviations: HC, high‐carbohydrate evening meal; HP, high‐protein evening meal; PSQI, Pittsburg Sleep Quality Index; ST, standard evening meal.

^a^ Changes within groups using repeated measures ANOVA.

^b^ Difference between groups using repeated measures ANOVA.

### 3.5 Biochemical measurements

Changes in laboratory measurements of the participants throughout the study are shown in Table 5. The inflammatory marker, hs‐CRP, decreased significantly in the ST and HC groups (*p* < .05), but it had no change in the HP group (*p* > .05). MDA reduction was not significant in any groups (*p* > .05). There were no significant differences between the groups for both hs‐CRP and MDA, by using both ITT and PP approaches (*p* > .05).

**TABLE 5:** Changes in hs‐CRP and MDA throughout the study

	ST (*n* = 36)				HC (*n* = 31)				HP (*n* = 29)				*p* value^b^
	Week 0	Week 10	Change	*p* value^a^	Week 0	Week 10	Change	*p* value^a^	Week 0	Week 10	Change	*p* value^a^	
hs‐CRP (µg/ml)	1.8 ± 1.8	1.3 ± 1.5	−0.5 ± 1.3	.03	1.7 ± 1.2	1.4 ± 1.3	−0.3 ± 0.7	.03	1.8 ± 1.5	1.8 ± 1.2	−0.01 ± 1.2	.98	.21
MDA (µmol/L)	2.27 ± 0.30	2.23 ± 0.20	−0.04 ± 0.31	.48	2.37 ± 0.29	2.27 ± 0.31	−0.10 ± 0.31	.08	2.30 ± 0.30	2.29 ± 0.33	−0.01 ± 0.34	.90	.52

Note

Values are mean ± *SD*.

Abbreviations: HC, high‐carbohydrate evening meal; HP, high‐protein evening meal; hs‐CRP, high‐sensitivity C‐reactive protein; MDA, malondialdehyde; ST, standard evening meal.

^a^ Changes within groups using paired *t* test.

^b^ Difference between groups using one‐way ANOVA.

## 4 DISCUSSION

### 4.1 Quality of life

The quality of life might be enhanced following a balanced diet and right eating habits in patients with type 2 diabetes (Davis et al., 2012; Shrestha & Ghimire, 2012). Studies have shown that weight loss and glycemic control may improve the quality of life in patients with type 2 diabetes (Porojan et al., 2009; Wycherley et al., 2014). In addition, a negative association has been found between physical health and systolic blood pressure (SBP), diastolic blood pressure (DBP), total cholesterol (TC), and low‐density lipoprotein cholesterol (LDL‐C) (Dehesh et al., 2019). In the present study, physical health significantly improved in the ST and HC groups, but not in the HP group. This finding may be due to less improvement in glycemic control, anthropometric measurements, and non‐significant lower improvement in the TC and SBP in the HP group, which was reported in our previous publication (Nouripour et al., 2021). In addition to weight loss and glycemic improvement, higher satisfaction with the diet in the ST group might be responsible for significant improvement of Mental Health component only in that group (Dehesh et al., 2019; Schnettler et al., 2015). However, there were no significant differences between the groups.

### 4.2 Sleep quality

In the present study, sleep quality had improved in all three groups. However, we did not observe any significant differences between the groups. In a crossover study by Yajima et al., the effect of high‐carbohydrate versus high‐fat meal on sleep structure was examined. They observed that high‐fat vs. high‐carbohydrate intake at dinner increases slow‐wave sleep during the first sleep cycle, which ultimately enhances subjective sleep quality (Yajima et al., 2014). In another crossover study using actigraph, a high‐protein diet for 4 days reduced wake‐ups during sleep compared with the control diet, while the high‐carbohydrate diet shortened sleep latency (Lindseth et al., 2013). In a similar study, a high‐carbohydrate diet resulted in shorter wake times compared with a high‐protein, high‐fat, or control diet (Lindseth & Murray, 2016). There is a lack of long‐term studies in this area.

It seems that amino acid tryptophan, the precursor of melatonin and serotonin synthesis, affects sleep quality. Therefore, increasing the food sources of tryptophan in the diet may improve sleep quality. Increasing the proportion of carbohydrates at the dinner increases postprandial insulin and may increase tryptophan entry to the brain and improve sleep (Binks et al., 2020).

Following items were frequently reported by our study participants regarding their sleep quality after initiation of the diet: decreased nocturia, more comfortable breathing, less snoring, and faster falling asleep. Respiratory disturbance is a factor that negatively affect sleep quality in patients with type 2 diabetes (Colbay et al., 2015). Weight reduction may improve obstructive sleep apnea and improve sleep quality (Tuomilehto et al., 2013). Mild weight loss following all three diets, which was reported previously, may explain parts of the findings in our study (Nouripour et al., 2021). A lack of significant improvement in the sleep disturbances component of the PSQI questionnaire in our trial may be due to the low sensitivity of this questionnaire in patients with type 2 diabetes. Therefore, designing a diabetes‐specific questionnaire is strongly recommended for future studies.

Another reason for improved sleep quality in the present study may be due to the reduced HbA_1_c following three diets, as stated previously (Nouripour et al., 2021). Enhanced glycemic control decreases nocturia and improves sleep quality (Chang et al., 2017; Yoda et al., 2015). Sleep quality may affect the quality of life (Luyster & Dunbar‐Jacob, 2011). Hence, it is important to consider the sleep quality in the treatment plans of patients with diabetes. Designing a trial with more differences between the diets is required.

### 4.3 Oxidative stress

As far as we know, this is the first study to compare the effect of carbohydrate and protein distribution among the meals on oxidative stress. The results of the present study did not show any positive effect of the prescribed diets on oxidative stress marker, MDA. This observation may be in part due to consistent intake of antioxidants throughout the study. In other words, the intake of α‐tocopherol, selenium, and beta‐carotene did not significantly change throughout the study (Vincent & Taylor, 2006). The only exception was ascorbic acid intake, which increased significantly in the HC group and it was accompanied by a non‐significant higher MDA reduction in that group.

The results of studies that had evaluated the effect of diet on oxidative stress and MDA level are controversial. In some studies on obese individuals with or without diabetes, a weight loss diet improved MDA level. However, others did not show beneficial effects (Crujeiras et al., 2006; Neyestani et al., 2004; Parra et al., 2007; Skrha et al., 2005; Wycherley et al., 2008). Further research is required to evaluate the effect of diet on MDA level in patients with type 2 diabetes.

### 4.4 Inflammation

The inflammatory marker hs‐CRP improved significantly in the ST and HC groups, which might be due to the direct effect of the prescribed diets and also weight loss and glycemic improvement subsequent to the diets as reported previously (Berk et al., 2016; Kalninova et al., 2014; King et al., 2003; Nouripour et al., 2021; Nowlin et al., 2012). However, hs‐CRP did not change significantly in the HP group, possibly due to less improvement in glycemic control or anthropometric measurements in this group, (Nouripour et al., 2021). In sofer et al. study on obese individuals, a hypocaloric diet with carbohydrates that was eaten mostly at dinner reduced tumor‐necrosis‐factor‐α (TNF‐α) and increased adiponectin concentrations in comparison to the control diet after 6 months of intervention (Sofer et al., 2011). Since adiponectin has an anti‐inflammatory effect, less improvement in hs‐CRP concentration in the HP group of our study might also be related to this factor (Van Dyke & Kornman, 2008). However, serum CRP did not significantly change in sofer et al. study (Sofer et al., 2011). Further studies are warranted in this area. We did not observe a significant difference between the three diets. This finding may be due to the hs‐CRP level of our participants which was in the normal range, so the variations were small.

### 4.5 Study limitations

In the present study, due to financial constraints, examination of inflammation and oxidative stress were only based on the hs‐CRP and MDA levels, respectively. Not much differences between the distribution of protein and carbohydrates among the meals were another limitation of this study. We were afraid that the higher differences between the diets would reduce the compliance dramatically.

## 5 CONCLUSION

Medical nutrition therapy might improve sleep quality in patients with type 2 diabetes. Minor manipulation of protein and carbohydrate distribution in the meals may not affect sleep quality. In addition, a diet with an even distribution of macronutrients among the meals or with a higher percentage of carbohydrates in the evening might improve the quality of life and reduce inflammation in patients with type 2 diabetes, while a diet with a higher percentage of protein in the evening may not improve it.

## CONFLICT OF INTEREST

None.

## ETHICAL APPROVAL

This study was conducted according to the Declaration of Helsinki guidelines and its later amendments, which was approved by the local Ethics Committee of Shiraz University of Medical Sciences (No. IR.SUMS.REC.1397.101). Written informed consent obtained from all study participants.

## Supporting information

Supplementary Material

## Data Availability

The data that support the findings of this study are available on request from the corresponding author.
