# Supplementary material for: Effect of protein and carbohydrate distribution among meals on quality of life, sleep quality, inflammation, and oxidative stress in patients with type 2 diabetes: A single‐blinded randomized controlled trial
Source: Food Sci Nutr. 2021 Sep 13;9(11):6176–85. doi: 10.1002/fsn3.2570 (PMC8565242; doi:10.1002/fsn3.2570)
Supplement: Supplementary file 1 — Supplementary Material [file FSN3-9-6176-s001.doc]

**Supplementary Material**

Effect of protein and carbohydrate distribution amongst meals on quality of life, sleep quality, inflammation, and oxidative stress in patients with type 2 diabetes: a single-blinded randomized controlled trial

**A sample 1800 Kcal ST diet**

**Breakfast:** 2 servings of bread and cereal + 1 serving of meat + 1 serving of vegetables + 1 serving of fat

**Morning snack:** 1 serving of fruit + 1 serving of milk + 1 serving of fat

**Lunch:** 4 servings of bread and cereal + 2 servings of meat + 1 serving of vegetables + 2 servings of fat

**Afternoon snack:** 1 serving of bread and cereal + 1 serving of fruit

**Dinner:** 3 servings of bread and cereal + 1 serving of meat + 1 serving of vegetables + ½ serving of milk + 2 servings of fat

**Evening snack:** 1 serving of fruit + ½ serving of milk

**A sample 1800 Kcal HC diet**

**Breakfast:** 2 servings of bread and cereal + 1 serving of meat + 1 serving of vegetables + 1 serving of fat

**Morning snack:** 1 serving of fruit + ½ serving of milk + 2 servings of fat

**Lunch:** 3 servings of bread and cereal + 2 servings of meat + 1 serving of vegetables + 2 servings of fat

**Afternoon snack:** 1 serving of bread and cereal + 1 serving of milk

**Dinner:** 4 servings of bread and cereal + 1 serving of meat + 1 serving of vegetables + ½ serving of milk + 1 serving of fat

**Evening snack:** 2 servings of fruit

**A sample 1800 Kcal HP diet**

**Breakfast:** 2 servings of bread and cereal + 1 serving of meat + 1 serving of vegetables + 1 serving of fat + ½ serving of milk

**Morning snack:** 1 serving of fruit + 1 serving of bread and cereal + 1 serving of fat

**Lunch:** 4 servings of bread and cereal + 1 serving of meat + 1 serving of vegetables + 3 servings of fat

**Afternoon snack:** 1 serving of bread and cereal + 1 serving of fruit

**Dinner:** 2 servings of bread and cereal + 2 servings of meat + 1 serving of vegetables + 1 serving of milk + 1 serving of fat

**Evening snack:** 1 serving of fruit + ½ serving of milk

**Servings:**

**1 serving of bread and cereal:** 30 g bread or 1/3 cup cooked rice or 1/2 cup spaghetti or 6 crackers or 1/2 medium boiled potato or 1/2 cup bread and starchy vegetables like corn or green peas or …

**1 serving of meat:** 30 g meat (or fish or chicken or turkey) or 1 medium egg or 30 g feta cheese or …

**1 serving of milk:** 1 cup low-fat milk or 2/3 cup low-fat yogurt or 2 cups doogh drink or …

**1 serving of fruit:** 1 small apple or 1 medium orange or 1 medium peach or …

**1 serving of vegetables:** 1 cup raw vegetables or 1/2 cup cooked vegetables or …

**1 serving of fat:** 1 tsp vegetable oil or 2 walnuts or 6 almonds or 10 peanuts or 1 Tbsp pumpkin or sunflower or sesame seeds or 9 large olives or …

**Note:** ½ cup cooked beans or peas or lentils = 1 serving of lean meat + 1 serving of bread and cereal

**Dietary recommendations:**

- Limit intake of foods with high sugar content like cola, jam, sweets, candies, commercial fruit juices, etc.
- Limit intake of high glycemic index foods like watermelon, white bread, etc.
- Eat legumes and soya at least two times per week.
- Eat whole grains instead of white bread or white rice.
- Consume fruits as a whole instead of fruit juices.
- Eat a variety of foods in amounts permitted in your diet.
- Use vegetable oils such as olive oil or canola.
- Avoid hydrogenated oils.
- Limit intake of trans fatty acid sources like cookies, ice cream, etc.
- Limit intake of foods with high saturated fatty acid like butter, cream, mayonnaise, etc.
- Eat lean meat, fish or poultry.
- Avoid fast foods like pizza, sausage, hamburger, etc.
- Consume low-fat dairies.
- Limit intake of fried foods. Instead, use boiled or steamed foods.
- Limit intake of salt and foods with high sodium content like pickles and processed foods.

Table 1. Changes in dietary intakes from weeks 0 to 10

|  | ST (n = 36) | HC (n = 31) | HP (n = 29) | P value^1^ |
| --- | --- | --- | --- | --- |
| Energy (Kcal) | -219 ± 337** | -283 ± 383*** | -297 ± 463** | 0.70 |
| Carbohydrate (g) | -42.6 ± 54.5*** | -48.2 ± 66.9*** | -44.7 ± 62.8*** | 0.93 |
| Carbohydrate (% of energy) | -2.4 ± 5.1** | -1.0 ± 6.2 | -0.6 ± 6.1 | 0.40 |
| Protein (g) | -0.8 ± 15.1 | -1.1 ± 16.7 | -2.3 ± 19.3 | 0.93 |
| Protein (% of energy) | 1.5 ± 2.2*** | 2.3 ± 2.6*** | 1.9 ± 2.2*** | 0.77 |
| Fat (g) | -5.3 ± 15.5* | -9.2 ± 20.3* | -12.9 ± 25.0* | 0.28 |
| Fat (% of energy) | 1.0 ± 4.5 | 0.3 ± 5.7 | 0.9 ± 5.1 | 0.85 |
| Fiber (g) | 1.0 ± 4.9 | 0.8 ± 7.8 | -1.6 ± 9.8 | 0.29 |
| α-tocopherol | -0.08 ± 0.63 | -0.08 ± 0.68 | 1.5 ± 8.1 | 0.28 |
| Selenium | -0.0005 ± 0.009 | 0.004 ± 0.01 | 0.0004 ± 0.001 | 0.27 |
| Beta-carotene | 26.4 ± 98.6 | 17.01 ± 264.9 | -36.7 ± 286.7 | 0.50 |
| Ascorbic acid | 6.8 ± 26.3 | 16.1 ± 29.7** | 41.4 ± 156.7 | 0.29 |

Values are mean ± SD.

Abbreviations: ST, standard evening meal; HC, high carbohydrate evening meal; HP, high protein evening meal.

* P < 0.05; ** P < 0.01; ***P < 0.001

^1^ Difference between groups using one-way ANOVA.

Table 2. Physical activity level of the participants throughout the study

|  | ST (n = 36) | HC (n = 31) | HP (n = 29) | P value^1^ |
| --- | --- | --- | --- | --- |
| IPAQ score (week 0) | 1296 ± 1313 | 1083 ± 1677 | 975 ± 1060 | 0.63 |
| IPAQ score (week 5) | 1280 ± 1303 | 1123 ± 1801 | 924 ± 1045 | 0.61 |
| IPAQ score (week 10) | 1237 ± 1201 | 1101 ± 1691 | 914 ± 1029 | 0.63 |

Values are mean ± SD.

Abbreviations: ST, standard evening meal; HC, high carbohydrate evening meal; HP, high protein evening meal; IPAQ, International Physical Activity Questionnaire.

^1^ Difference between groups using one-way ANOVA.
